# Patient Choice for Older People in English NHS Primary Care: Theory and Practice

**DOI:** 10.1155/2014/742676

**Published:** 2014-03-04

**Authors:** Andrew J. E. Harding, Frances Sanders, Antonieta Medina Lara, Edwin R. van Teijlingen, Cate Wood, Di Galpin, Sue Baron, Sam Crowe, Sheetal Sharma

**Affiliations:** ^1^School of Health & Social Care, Bournemouth University, Bournemouth House, 19 Christchurch Road, Bournemouth, Dorset BH1 3LH, UK; ^2^Westbourne Medical Centre, Milburn Road, Bournemouth, Dorset BH4 9HJ, UK; ^3^Health Economics Group, University of Exeter Medical School, Veysey Building, Salmon Pool Lane, Exeter EX2 4SG, UK; ^4^Plymouth University, Drake Circus, Plymouth, Devon PL4 8AA, UK; ^5^Dorset County Council, Vespasian House, Bridport Road, Dorchester DT1 1TS, UK

## Abstract

In the English National Health Service (NHS), patients are now expected to choose the time and place of treatment and even choose the actual treatment. However, the theory on which patient choice is based and the implementation of patient choice are controversial. There is evidence to indicate that attitudes and abilities to make choices are relatively sophisticated and not as straightforward as policy developments suggest. In addition, and surprisingly, there is little research on whether making individual choices about care is regarded as a priority by the largest NHS patient group and the single largest group for most GPs—older people. This conceptual paper examines the theory of patient choice concerning accessing and engaging with healthcare provision and reviews existing evidence on older people and patient choice in primary care.

## 1. Introduction

Market reforms in the English National Health Service (NHS) have been implemented to make services more responsive [1]. Since the internal NHS market reforms of the late 1980s, recent policies have directly and indirectly encouraged the entry of alternative third and private sector providers [2]. These reforms position patients as consumers who (a) make choices instead of their medical and healthcare professionals and (b) are increasingly expected to navigate local health economies. Patients are now expected to choose a time and place of treatment [3], and even choose a treatment [2]. In this consumer revolution patients are now to take responsibility for the choices that they make. The 2010 Coalition Government White Paper stated that

*“In return for greater choice and control, patients should accept responsibility for the choices they make.” [2].*

But is this realistic? In a healthcare setting, is the best treatment a choice? do patients want to make a choice? and do patients really make choices? It is likely that the answers to these questions will differ depending on medical context and social group. However, the theory on which patient choice is based and the implementation of patient choice are controversial [1, 4–8]. There is evidence to suggest that attitudes and abilities to making choices are relatively sophisticated and not as straightforward as policy makers suggest [9]. In addition, patient choice is a hugely under-researched area [10]. Surprisingly, there has been little research on whether making individual choices about care is regarded as a priority by the largest NHS patient group.

Due to diseases associated with ageing and the increased prevalence of long term and chronic conditions, older people (defined as being over 65 years of age) are the largest NHS patient group in general and in primary care in particular [11]. Despite this, our knowledge of how older patients make choices and the extent to which patients are prepared to become involved in healthcare decision making is limited. This paper examines the theory of patient choice as a means of accessing and engaging with healthcare provision and reviews existing evidence and research on older people and patient choice in primary care.

## 2. Theory

A general practitioner (GP) referral is the most common gateway into secondary care. Primary care is also the medical setting where the initial healthcare decisions and choices are made. The assumption behind placing patient choice as the key mechanism toward making services increasingly responsive is clear. As consumers, patients are regarded as being able and willing to navigate healthcare provision by making informed and often complex choices about treatment pathways [12–14].

Theoretically, these choices about providers and treatments should be evidence based and rational and correlate to need so that underperforming and substandard providers either improve their performance to attract patients or exit the market.

Yet, with evidence that greater patient involvement in care has beneficial outcomes [15, 16], and that involvement in decision making is consistently high up the patient and public agenda [9, 17, 18], this set of assumptions and the underpinning theoretical proposition is laudable.

However, counter theoretical propositions that pose significant problems for positioning the patient, and especially the older person, as a healthcare consumer exist [1, 4, 8, 19].

## 3. Patients Are Different from Consumers in Other Markets

Many have argued that choosing between healthcare providers and treatments is unlike any other service or product [1, 4, 20, 21]. When compared to many other consumer products, in healthcare, there is a pressing need to consult expertise in order access provision. In economic and market theory the principal agent relationship is used to describe a situation when information is complex and asymmetric and in order to become informed, an individual (principal) consults the expertise of another (agent). In a medical context this can be seen when a patient (principal) consults a doctor (agent). If the expertise is held by doctors, they are the dominant actor in the patient-doctor relationship. There is a comprehensive literature on the nature of this type of agency which underlines the complexity of accessing welfare provision, and the need to consult expertise in order to become informed [19, 21, 22].

Problems of applying a consumer choice model to healthcare become apparent when consideration is given to the conditions needed for patients to exit when dissatisfied. In order to exit, at the very least, a patient will require awareness that treatment is poor. In some instances this may require intimate knowledge of the medical condition, and even when it may not require this knowledge, delineating poor levels of treatment may not always be easy to identify. There are obvious exceptions, for example, instances of abuse and neglect. However, on the whole, it is not unreasonable to suggest that patients may not know if their treatment is poor. Indeed, in most cases it is of little use being able to delineate if treatment is poor, if you are unaware of the quality of alternative providers. On this basis, being able to exit will often require an intimate knowledge of the medical condition and knowledge of the clinical quality of alternative providers is known. Unsurprisingly, the opinion of friends and family has been found to be a much more popular source of information when choosing a provider than more formal information, for example, on the relative clinical quality of providers [23]. On this basis, patients have been suggested to be flawed consumers who at no stage are able to make informed choices [4]. In other words, consumers are in no position to be sovereign and dictate the quality of producer's goods and services [24].

Considering the conditions required for patients to exit underlines that healthcare is an excellent example of the principal agent dynamic. However, positioning patients as responsible consumers fundamentally questions this agency dynamic and the need for it. Responsible implies good, and a good choice is based on understanding complex information. Indeed if patients are making autonomous choices, they would effectively assume the role of the principal and the agent, instead of the medical profession. Although the desired level of autonomy and extent to which patients are willing to make independent choices is unclear, it is hard to envisage a good patient choice being made without the help of a doctor. On this basis, the use of the word “choice” seems disingenuous and misleading. Patients will very rarely have a “free” choice without needing support. Rather, as patients require medical professionals in order to become informed, it seems more accurate to refer to the choice process as one where patients are enabled to be involved in decision making. In order to illustrate the complexity need to make healthcare choices, it is important to discuss what the types of choices that will need to be made.

## 4. Choosing What—Provider or Care?

Some patient choice literature and research tend to draw a line between having to make a choice of provider and treatment [17, 25]. However, whilst deciding* when* to be treated could be considered a fairly mundane choice focused on convenience [1], there are many instances where provider and treatment choices are linked. Being able to delineate between the performances of different providers suggests that there are cases where a choice of provider will be more complex than choosing a convenient time. A good patient choice will need to be based on information about the performance of all available providers (competitors) and the success rates for different treatments. In order to demonstrate the complexity of this type of data, it is worth considering how to interpret data for success rates. Firstly, it is valid to pose the question “What constitutes success?”. Large data sets are only likely to report on providers' success rates and possibly hide more useful information on different levels of clinical need. For example, since success rates are affected by case mix, providers with proportionately more poor patients have poorer outcomes. This type of data is relatively inaccessible and asymmetric and tends to be intimidating [26], and interpretation requires experienced medical professionals. Instead and unsurprisingly, studies have indicated that it is common for patients to make choices based on less formal information that tends not to provide clinical information. For example, feedback from family members, the local press [7, 23], the accessibility, and quality of car parking facilities [1, 27] have all been cited as common sources of information that patients consult in order to make medical choices. In other words, often, “…patients are extremely flawed consumers” [4]. There is also supported evidence from the personalisation agenda in social care. When social service support is substituted for a direct payment or individual budget, older people operating as consumers by making choices have been found to be associated with poor psychosocial outcomes [28]. On this basis, patients in a primary care setting are likely to defer to medical opinion and therefore lack the willingness and the ability of an active and informed consumer [1, 4, 7, 8, 21, 22, 29].

Conversely, it has been suggested that certain patient groups will likely gain expertise on account of accessing and engaging with healthcare on a frequent basis [1]. This seems more plausible for older patients who, on account of having a higher prevalence of long term and chronic conditions, will tend to access provision much more frequently, and perhaps gain relative expertise. However, there is little research evidence to support this. What little evidence that does exist, for example, from the “Expert Patients Programme” does not examine whether patients who underwent the programme made more choices. Rather, studies actually indicate that expert patients were not as expert as envisaged [30, 31].

The theoretical assumptions in this discussion are just that—assumptions. The following section examines the evidence regarding these issues in English primary care. The UK population, including older people, has continually expressed an interest in becoming involved in the decision making that affects their healthcare [9, 15, 17, 18, 29, 32, 33]. Yet, the existing research on older people and patient choice in primary care suggests that preferences and views about choice are more sophisticated and not fully encompassed by a simple desire to make choices.

## 5. Evidence and Practice: Patients Making Choices

Exercising choice was found to be high during the London Patient Choice Project [13]. However, this was conducted on patients who were on a long waiting list and were offered subsided transport costs on London's extensive transport network and supported by advisors in making choices [4]. This indicates that people will shop around if local providers encounter poor outcomes in areas such as London with many providers in a small geographical area. However, evaluations of the London Patient Choice Project indicate that older people (aged over 60) were less likely to choose an alternative provider when offered [34]. This finding, or a preference not to shop around, is supported in other studies.

Two qualitative research studies underline that older people do wish to make a choice of provider in a GP consultation [9, 25]. However, both studies reveal that attitudes are much more complex; on the one hand, people see the value in being able to choose, on the other hand, they tend to choose their local provider. They prefer a local provider who is familiar and convenient and offers easy access [9, 25], and there is even evidence that local providers are preferred when a better medical alternative is offered. For example, a local study with stroke patients in Dorset found that having access to hospital care that was easier for friends and family to access in order to maintain social networks is often more important than travelling a longer and more costly distances, even when it is likely that the service would offer better clinical outcomes [35]. In addition, user experience or quality of service has been found to have more value for patients than clinical effectiveness [36].

A tendency to prefer local and convenient providers is consistent with other research, and not exclusive to older people [7, 23], or even older people in the UK [33]. Generally, a choice of healthcare provider may be desirable, but for older people it does not seem to be a priority.

For older patients in England, Weir et al.'s [9] findings may provide a reason as to why making a choice of provider is regarded as relatively unimportant by older people. The mechanism that allows patients in England to choose a provider for an elective referral in primary care is the online based “choose and book” system [37]. Yet, due to a lack of engagement with information technology (IT), older people have suggested that having to access the internet to “choose and book” is inappropriate and leads to “rushed” choices. Unsurprisingly, most older people would prefer access to more traditional and conventional literature [9]. A lack of engagement with IT could also explain why a broader study found that GPs, with or without “choose and book,” often tend to make referrals on patients behalf [27]. It is unclear whether a more suitable choice of provider mechanism would encourage older people to shop around or, whether generally, older people will always be relatively indifferent to choosing a provider that is not local.

In terms of making choices and becoming involved in treatment decisions, Weir et al. [9] found that older people think that “the GP knows what is best for you in your situation and condition.” With a lack of preexisting knowledge, this finding is not surprising [38]. However, Weir et al. [9] also find that this view does not equate to a complete deferral of decision making to GPs. The view that “the GP knows what is best…” was often found to be consistent with a preference for decisions to be made in partnership with a GP. A cross European study indicates that older people appreciate qualitative information about their health condition so that treatment options can be discussed, with opportunities to ask questions and be listened to [33], and this is consistent with Weir et al.'s [9] English study. However, in reality the participants in Weir et al.'s [9] study stated that GPs often did not regard them as being able to make choices through reinforcing ageist stereotypes (e.g., through being hard of hearing or taking longer to process information) and often assumed they could not or did not permit them enough time to do so. Some have recommended extending the time for consultations where referrals and choices need to be made [23], and as Weir et al. [9] suggested, this seems especially relevant to the elderly.

Another broader qualitative study that elicits GP views of patient choice provided some reasons as to why practice may not always enable patient choice, control, and responsibility [27]. They found that support among GPs for facilitating patient choice is hugely diverse. The study, which canvassed the views of GPs from a broad range of localities, categorised participants as enthusiasts, sceptics, or paternalists. Many were either sceptical of the value of choice and/or thought that they knew best. Rosen et al's [27] study indicated that many GPs agreed with the kind of concerns discussed in the theoretical section of this paper; namely, that the ability to form a rational choice based on understanding “formal” information is often not realistic. Yet, as established, this is not consistent with what previous studies have indicated about older people; namely, that they wish to be enabled to become involved in decision making [9, 33].

There are, of course, structural and organization constraints when offering choice, especially choice of provider. Rosen et al. [27] found that GPs were more enthusiastic and had experience of facilitating choice in localities where a choice of provider exists (e.g., in inner cities, such as London, that have more providers and better transport links). Whilst, on the other hand, GPs were less enthusiastic and had little experience of facilitating choice in areas with only one regional hospital. On enabling patients to have greater involvement, with limited budgets and resources, a significant finding was that GPs had fears that supporting choice in some patient groups may incur extra costs. Potential extra costs might include the extra time required to help patients obtain and understand information and the potential cost of employing “choice advisors.” Le Grand, a key architect of patient choice policies [12–14, 39], recommends the introduction of “patient choice advisors” in his evaluation of the London Patient Choice Project [13]. Interestingly, a proposed need to recruit advisors goes against the assumption that patients are informed and rational consumers, and instead reinforces the need for principal agent theory and that in current practice it is not appropriate to label patients, including older patients, as consumers.

## 6. The Way Forward

This paper reviewed theoretical issues and the application in practice of patient choice policies in relation to older people in primary care. There is evidence to indicate that, overall, older people are relatively happy with the NHS. A recent British Social Attitude report indicates that three-quarters of the British population over the age of 75 are satisfied with the NHS [40]. However, perhaps the evidence reviewed in this paper would suggest that satisfaction would be much lower if the focus was on involvement in decision making in primary care. Indeed, as discussed, Coulter [18] indicates that not being involved in decision making is the biggest cause of patient dissatisfaction in the NHS. But what is possible to conclude from the research on older people and patient choice in primary care?

There is some evidence to suggest that older people wish to become enabled to become involved in healthcare decision making in partnership with a GP (thus, reinforcing principal agent theory), but that this is often not realised. Instead, research indicates that GPs often assume older people lack the capacity or the desire to innovate in order for older people to become involved in decision making. However, with a relatively small evidence base, it is not clear what the dominant factors are in older people not being enabled to become involved in healthcare decision making. Whilst it is not possible to generalize from this small evidence base and categorically state that general practice in England does not enable older people to become involved in healthcare decision making, recent high profile reports indicate that this issue is an important one to consider.

In their interactions with older people, GPs have a key role in making information accessible and ensuring that older people are involved in healthcare decisions.* The Francis Report* has recently reemphasised the need for GPs (as practitioners* and now commissioners* in England) to make choice a reality for patients [41]. Whilst this will no doubt mean increasing the amount of providers, being knowledgeable of their services and developing a greater understanding of the factors that affect where patients choose to be treated, it will also require innovation, established guidance, and recommendations as to how practice can be developed to become flexible enough to fully facilitate older people in becoming involved in healthcare decision making. For example, two possibilities that emerge from the research literature could be additional material and physical support for older people and extended consultation times. Such measures, and others, would allow much more collaborative decision making so that decisions would be made in partnership with empowered older people.

The evidence reviewed in this paper indicates that policy makers, practitioners, and academics need to give consideration as to how to better configure GP services to enable older people, as the largest patient group, to become involved in healthcare decision making.
